# Supplementary material for: Optical-resolution functional gastrointestinal photoacoustic endoscopy based on optical heterodyne detection of ultrasound
Source: Nat Commun. 2022 Dec 9;13:7604. doi: 10.1038/s41467-022-35259-5 (PMC9734171; doi:10.1038/s41467-022-35259-5)
Supplement: Supplementary file 1 — supplementary information [file 41467_2022_35259_MOESM1_ESM.pdf]

Supplementary Information:

Optical-resolution functional gastrointestinal photoacoustic endoscopy based on optical heterodyne detection of ultrasound

Liang et al.

Supplementary Note S1 Heterodyne phase detection and noise characterization.

Supplementary Note S2 Ultrasound response.

Supplementary Note S3 Comparative study: Optical sensors versus piezoelectric sensors.

Supplementary Note S4 Error analysis of sO_2 measurement.

Supplementary Note S5 PAE.

Supplementary Figure S1 Schematic and noise characterization of the ultrasound sensing system.

Supplementary Figure S2 Ultrasound response.

Supplementary Figure S3 Stability test result of the sensor.

Supplementary Figure S4 Difference in the working mechanism between piezoelectric and optical ultrasound sensors.

Supplementary Figure S5 NEPDs of piezoelectric and optical ultrasound sensors versus diameter.

Supplementary Figure S6 Photoacoustic measurement of sO_2.

Supplementary Figure S7. Fabrication of the endoscopic probe.

Supplementary Figure S8 PAE system.

Supplementary Table S1 Ultrasound sensitivities and energy transduction ratios of piezoelectric and optical sensors.

Supplementary Note S1: Heterodyne phase detection and noise characterization.

Figure S1a shows a schematic of the ultrasound sensing system. The sensing element is a laser cavity confined by two Bragg gratings fabricated in a rare-earth-doped fiber. The sensor laser has a single-longitudinal-mode laser output with both x- and y-polarizations when pumped with a 980-nm semiconductor laser through a wavelength-division multiplexer (WDM). The orthogonal laser beams have slightly different lasing frequencies due to the intrinsic birefringence. Figure S1b exhibits the measured optical spectrum of the laser output with a frequency resolution of 10 MHz (optical spectrum analyzer: BOSA 200 CL, Aragon Photonics Labs), which shows a $\omega_b = 2\pi \times 1.739$ GHz frequency spacing. An optical isolator is used to prevent unwanted optical scattering or endface reflection into the cavity. The x- and y-polarized laser light beams heterodyne at the InGaAs photodetector (DSC50S, Discovery Semiconductors, Inc.), producing a radio-frequency beat note at 1.739 GHz. A polarizer and a polarization controller are used to maximize the beat signal. We amplify the laser output from 0.5 mW to 20 mW with an EDFA (specialized by Beogold Technology, China) to suppress the shot noise at the photodetector. We then measure the beat signal using an electrical spectrum analyzer with a resolution of 10 kHz. The measured power spectrum in Fig. S1c presents two sidebands with a frequency offset of 1.7 MHz due to laser relaxation oscillation, one of the prominent types of intensity noise of fiber lasers.

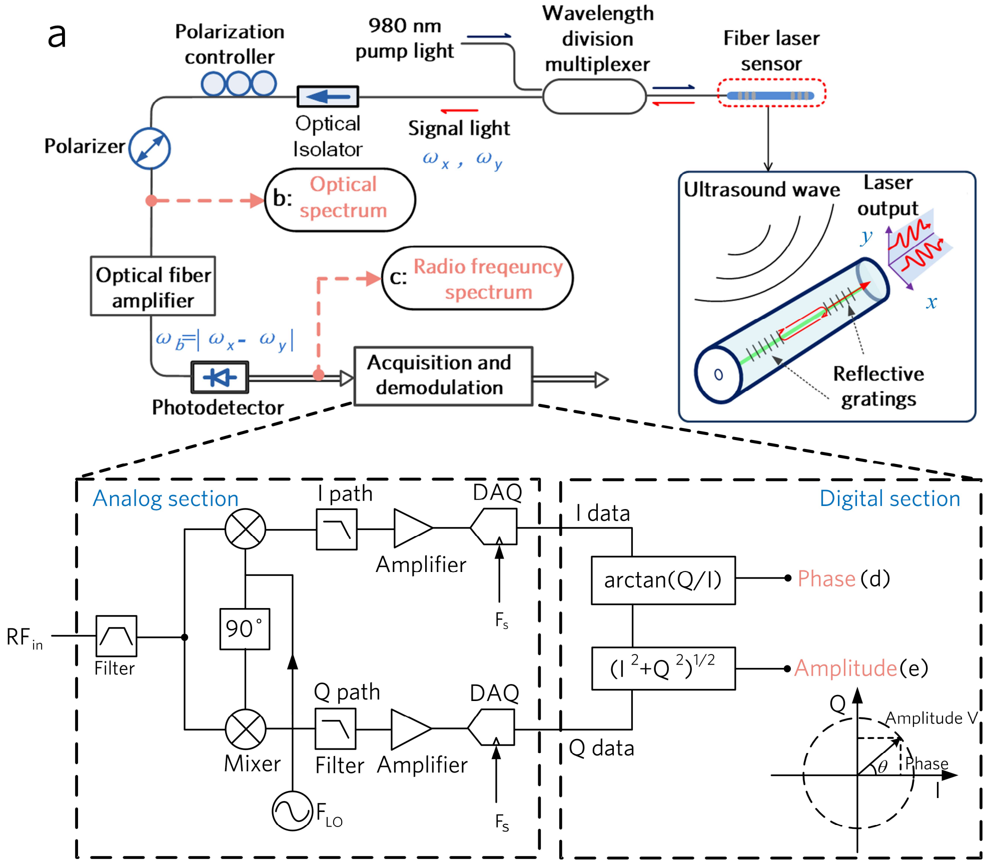

(to be continued)

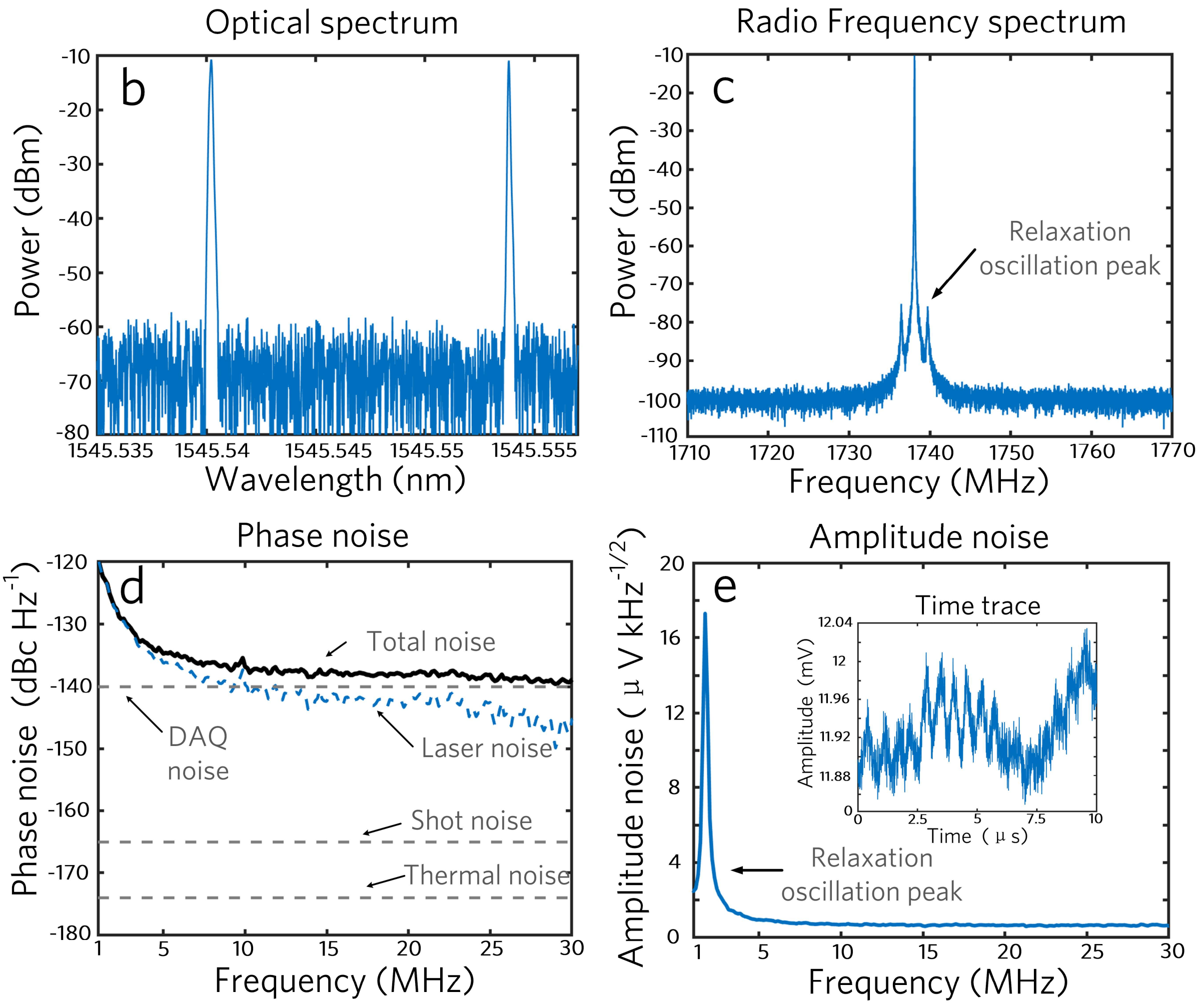

Supplementary Fig. S1. Schematic and noise characterization of the ultrasound sensing system. (a) Configuration of the sensing system. Upper inset: laser sensor. Lower inset: I/Q demodulator. (b) Measured optical spectrum of the laser sensor. (c) Measured power spectrum of the radio-frequency heterodyne signal. (d) Phase and (e) amplitude noise spectra of the heterodyne signal. Inset of (e): Extracted time trace of amplitude fluctuations. The measured results in (c) and (e) show noticeable amplitude fluctuations induced by laser relaxation oscillation at 1.7 MHz. However, this noise was not coupled into phase demodulation, as shown in (c). The ports where the individual results were measured are marked in (a).

Notably, the laser linewidths measured by the optical and electrical spectrum analyzers cannot accurately characterize the noise properties. For precise noise characterization, we performed *I*/*Q* demodulation by using a vectorial signal transmitter (5646R, NI), as illustrated in the inset of Fig. S1a. The incoming radio-frequency signal was mixed with a local oscillator with a working frequency close to ω_b in the analog section to downshift the carrier frequency to baseband. The signal was then split into in-phase (*I*) and quadrature-phase (*Q*) components with a 90-degree offset, which were separately digitized with a field-programmable gate array (FPGA) after filtering

and amplification. We acquired the phase of the signal by calculating the arctangent of the ratio between I and Q data via arctan(Q/I) [1]. Figure S1d shows the phase noise spectrum with a DAQ noise at approximately -140 dBc/Hz. Its power density slowly decreases from approximately -130 dBc/Hz at 3 MHz to -140 dBc/Hz at 30 MHz. By subtracting the total noise from the DAQ noise, we can obtain the noise of the laser light after optical amplification. The average power density is approximately -140 dBc/Hz, with a descending profile with frequency. Spontaneous emission was reported to be the dominant noise source for fiber Bragg grating lasers at frequencies above 1 MHz [2]. It has a fundamental quantum limit known as the Schawlow–Townes relation [2, 3]. The contributions of our sensor laser and the EDFA can be written together as [4]

$$\langle N_{\mathrm{sig-s}} \rangle^2 \approx -155.9\ [\mathrm{dBm/Hz}] + NF\ [\mathrm{dB}] - P_{sig}[\mathrm{dBm}] \quad \text{(S-1)}$$

We boosted the optical intensity to approach the saturation limit at the photodetector, yielding a photocurrent of i_{dc}=10 mA, the $P_{sig} = -10$ dBm. The calculated $N_{\mathrm{sig\text{-}sp}}$ is approximately -142 dBc/Hz with a noise figure NF=4 dB, which agrees with the measured result, as shown in Fig. S1d.

Additionally, the thermal noise N_{oth} and shot noise N_{shot} at the photodetector can be expressed as [4]

$$\langle N_{\mathrm{oth}} \rangle^2 \approx \frac{k_{\mathrm{B}}T}{i_{\mathrm{dc}}^2 Z_{\mathrm{out}}} \approx -155\ [\mathrm{dBm/Hz}] - 20\log(i_{dc}[\mathrm{mA}])\ \ (matched\ 50\Omega\ load) \quad \text{(S-2)}$$

$$\langle N_{\mathrm{shot}} \rangle^2 \approx \frac{2e}{i_{\mathrm{dc}} Z_{\mathrm{out}}} \approx -155\ [\mathrm{dBm/Hz}] - 10\log(i_{\mathrm{dc}}[\mathrm{mA}]) \quad \text{(S-3)}$$

where $i_{\mathrm{dc}} = I_0 \cdot H_{\mathrm{pd}}$, with $H_{\mathrm{pd}} \approx 0.6\sim0.8$ A/W being the optical-to-electrical transduction coefficient, e denotes the elementary charge. The calculated values are $\langle N_{\mathrm{oth}} \rangle^2 = -175$ dBc/Hz and $\langle N_{\mathrm{shot}} \rangle^2 = -165$ dBc/Hz. In comparison, the contribution of the photodetector to the noise is minimal and was neglected.

Figure S1e shows the measured amplitude noise spectrum, obtained by extracting the amplitude of the beat signal with $V = \sqrt{I^2 + Q^2}$. Its time trace in the inset of Fig. S1e shows a noticeable periodic intensity fluctuation, which causes a peak in the amplitude noise spectrum. However, this peak is not visible in the phase noise spectrum because of the low coupling efficiency between the intensity noise and phase noise (1~3%) [2]. In addition, the residual transduced noise was eliminated via common-mode cancelation between the dual-frequency laser light beams. This comparison demonstrates that our sensing system is immune to intensity fluctuations. As a result, only the phase noise N_{q} limits the acoustic sensitivity.

Supplementary Note S2: Ultrasound response.

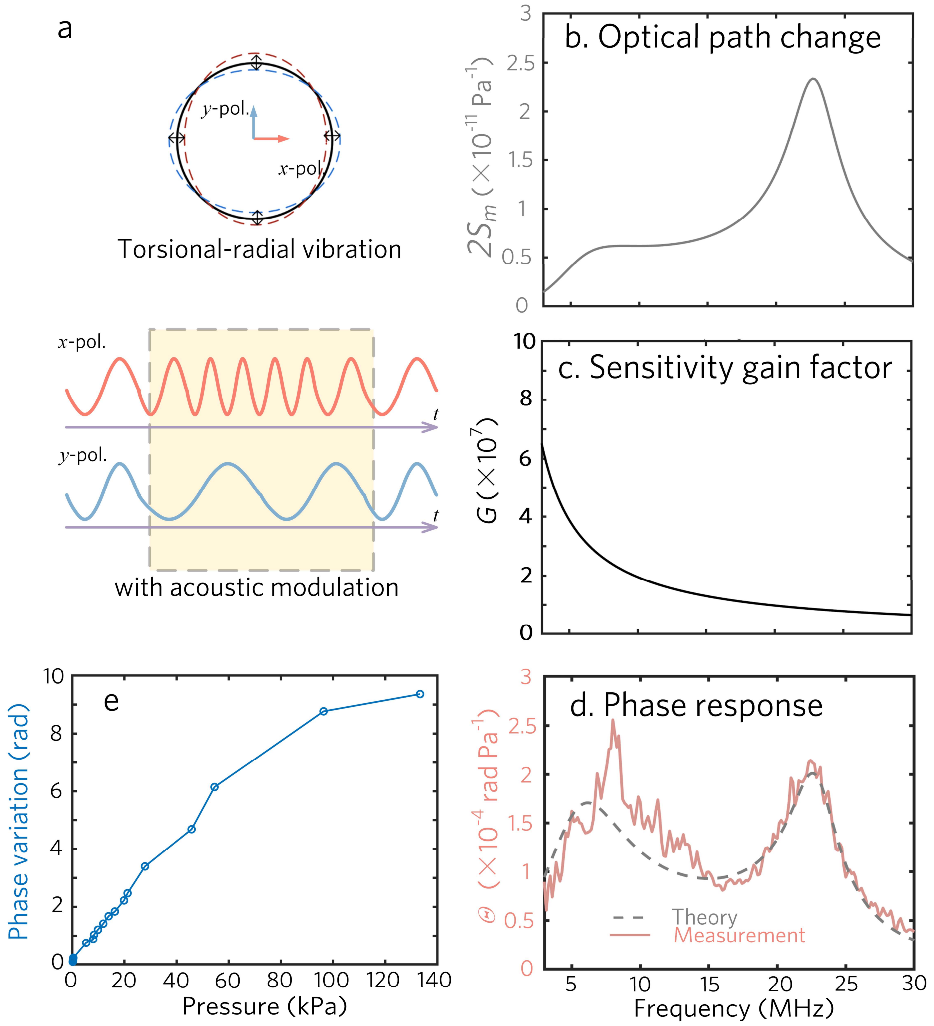

Supplementary Fig. S2. Ultrasound response. (a) Torsional-radial vibration. This vibration induces opposite phase changes in the orthogonally polarized laser beams and can be detected via heterodyne phase demodulation. (b)-(d) Frequency responses of the sensor geometry change (b), sensitivity gain factor (c), and phase variation (d). (e) Measured ultrasonically induced phase change as a function of applied acoustic pressure.

In this note, we calculate the ultrasound response $\Theta(\Omega)$. A bare optical fiber can be treated as a homogeneous elastic cylinder in acoustics. As shown in Fig. S2a, a torsional-radial vibration can be excited by an ultrasound wave and induce optical phase changes in the x- and y-polarized laser light beams. In the calculation, the longitudinal and shear waves in the silica fiber and the pressure waves in the surrounding water can be expressed by scalar and vectorial potentials, respectively. They are written in the Bessel functions of the first kind J_l and N_l, where l denotes the azimuthal mode order. Here, only l=2 components are responsible for the torsional-radial modes. We used three linear equations to describe the acoustic interaction between the fiber and surrounding water based on the continuity conditions of the acoustic stresses and displacements[5]. The

displacement/stress in the fiber can be obtained by solving the equations with a known incoming pressure wave. We can further calculate the acoustically induced optical response based on the photoelastic effect. Figure S2b shows the calculated frequency response $S_m(\Omega)$ of the optical path change to a planar ultrasound wave. The maximal response at $\Omega_{\text{res}}=2\pi\times22$ MHz is a result of the vibrational resonance. The resonance is relatively weak, with a quality factor of only 3 due to the strong dampening effect of the surrounding water. For ultrasound frequencies below 10 MHz, the acoustic wavelength becomes significantly larger than the fiber diameter, and the damping rate substantially decreases. As a result, the response curve $S_m(\Omega)$ deviates from an ideal Lorentz profile, yielding an asymmetric frequency response, as shown in Fig. S2b. The parameters used for the calculation were silica density ρ_s=2240 kg·m^{-3}, longitudinal wave velocity c_L=5878 m/s, shear wave velocity c_S=3706 m/s, water density = 1000 kg·m^{-3}, acoustic velocity in water c_w=1480 m/s, effective strain-optic coefficient p_{44}=-0.0695, and refractive index of silica glass n_0=1.45. The effect of fiber elongation is minimal and can be neglected here.

The torsional-radial vibrational mode compresses and stretches the fiber core in the two orthogonal directions. Therefore, for an in-core laser, this vibration induces equal phase changes of the orthogonally polarized laser light beams but with opposite signs (or a π phase offset). As a result, the acoustic modulation is doubled in heterodyne phase detection, and the output signal can be written as $\Theta(\Omega) = 2S_m(\Omega) \cdot G(\Omega)$. By multiplying $2S_m(\Omega)$ by the optical gain factor $G(\Omega)$ (the profile is shown in Fig. S2c), we can obtain the calculated phase response in Fig. S2d, which agrees with the measured result. The additional peak at approximately 8 MHz results from the sensitivity gain effect of $G(\Omega)$ because it amplifies the acoustic response more significantly at lower frequencies.

We then investigate the linearity range of the sensor output. Eq. (1) can be decomposed as an expansion of Bessel functions $v_s = \sum_{n=-\infty}^{+\infty} J_n(p_0 S_m G) \exp[i(\omega_0 + n\Omega)t]$. It indicates that a strong acoustic modulation produces multiple sidebands at $\omega_0 + n\Omega$. Linear response requires a large detection bandwidth to collect more sidebands to reconstruct the modulated signal [1]. Figure S2e shows the measured phase variation as a function of applied acoustic pressure. The result suggests good linearity to approximately 50 kPa (or a phase variation of 5 rad), using an acquisition bandwidth of 200 MHz.

The stability of the sensor output is vital for *in vivo* endoscopic imaging. Here, we performed a stability test with the experimental setup shown in Fig. S3a. We used an unfocused piezoelectric transducer (V216, Olympus) as an acoustic source, which emits ultrasound pulses at a repetition rate of 2 kHz. The sensor was immersed in water to continuously receive applied ultrasound waves. We recorded the sensor output under thermal, vibrational, and rotational perturbations and plotted the phase change induced by each ultrasound wave pulse to characterize the sensor stability. Figure S3b shows the sensor output with applied thermal drift. The water temperature varied from 33 to 43 °C, covering the average endotherm body temperature range. The measured acoustic modulation is highly stable, although the carrier frequency of the beat signal ω_b changes at a rate of -1.2 MHz/°C (not shown in the figure). Figure S3c shows the bending test result. The pigtailed fiber was mounted on a motorized linear stage to bend the optical fiber. The stage moved with a maximal speed of 4 mm/s. We repeated the back-and-forth scanning 1800 times in 30 min. The result shows a stable detection sensitivity, because the optical polarization state in the fiber almost remains unchanged during bending. Figure S3d shows the test result when the sensor was rotated back and forth for 45 min. The sensor was rotated clockwise by 90 degrees and then back to the

original angle to complete a scanning cycle. The rotational scanning was repeated at a rate of 1 Hz. The sensor has a maximum output when the principal axis is in accordance with the ultrasound incident direction (corresponding to 50 degrees in the left panel of Fig. S3d). When the applied ultrasound is incident at 45 degrees to the principal axis, the sensor has a minimal output (corresponding to 5 and 105 degrees in the left panel, Fig. S3d). The angle-dependent sensor output of each cycle is plotted in the middle panel of Fig. S3d. The right panel of Fig. S3d shows excellent sensor stability based on the extraction of the acoustically induced phase change at a certain angle over time. The test results suggest that the laser ultrasound sensor can effectively cancel the effect of thermal drift or other low-frequency perturbations via heterodyne detection by using highly correlated *x*- and *y*-polarized laser beams.

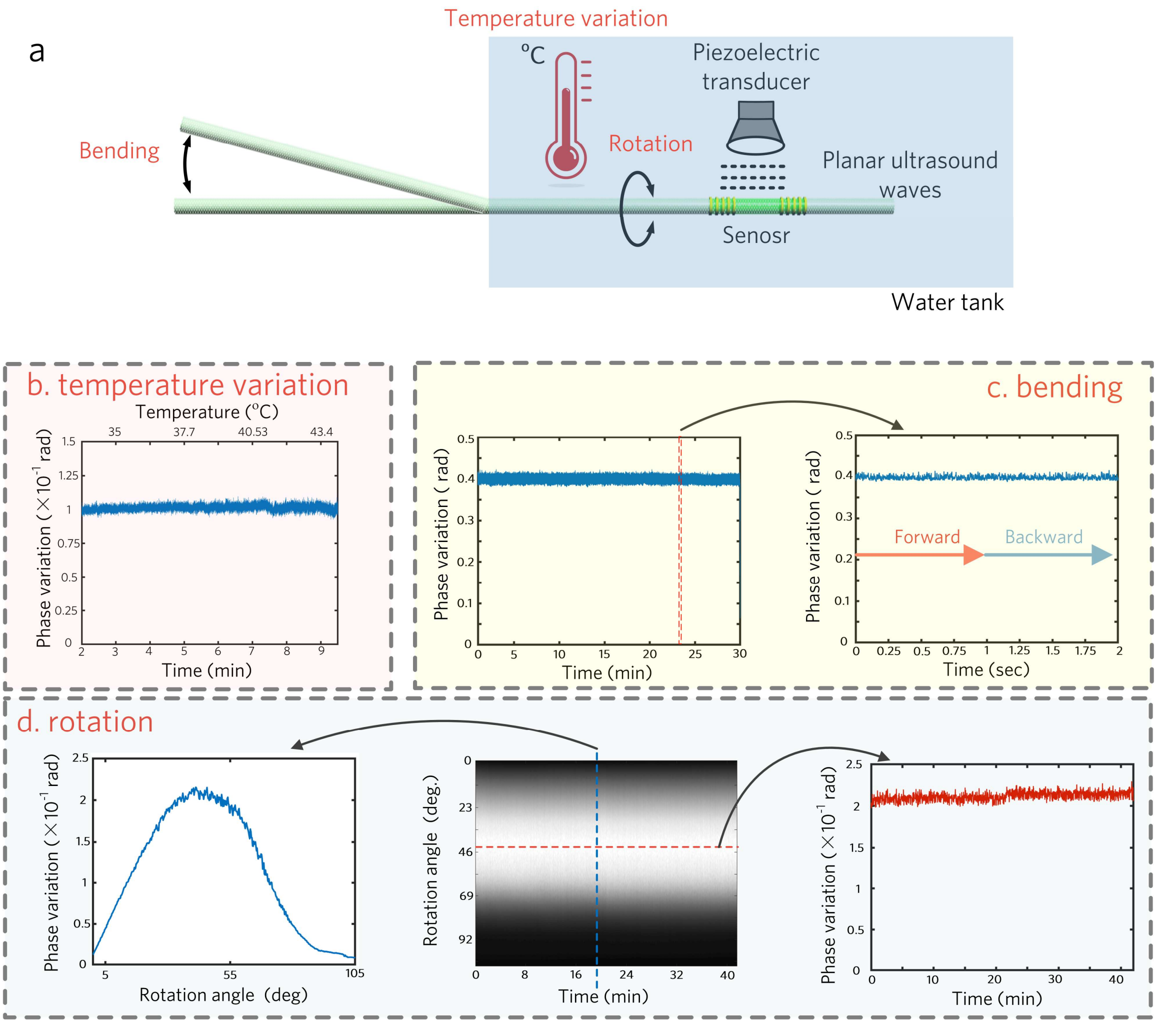

Supplementary Fig. S3. Stability test results of the sensor. (a) Experimental setup for the test. (b), (c), and (d) Test results with applied temperature variation, repeated fiber bending, and back-and-forth rotational scanning, respectively.

Supplementary Note S3 Comparative study: Optical sensors versus piezoelectric sensors.

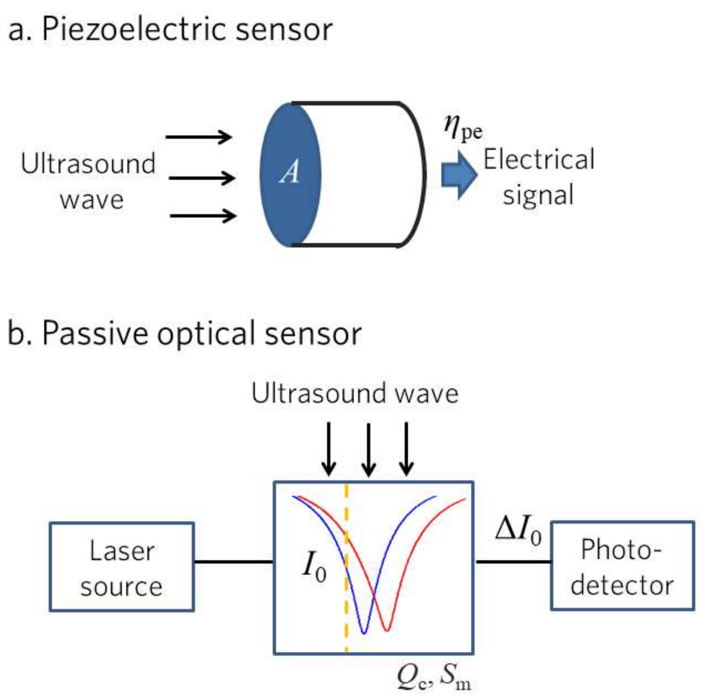

Supplementary Fig. S4. Difference in the working mechanism between piezoelectric and optical ultrasound sensors. (a) A piezoelectric sensor converts acoustic energy into electrical energy with a ratio η. The sensitivity can be scaled up by increasing the sensitive area A. (b) A resonator-based optical sensor transduces acoustic pressure into a modulation in the intensity I_0. The sensitivity is determined by S_m, which measures the sensor geometry modulation, and the quality factor Q_c, which amplifies the acoustic response.

In this note, we investigate the different mechanisms between piezoelectric and optical detection by examining the acoustic-to-electrical energy transduction.

A. Piezoelectric sensors

A piezoelectric sensor partially converts the acoustic energy of an exerted pressure wave into electrical energy (Fig. S4a). The acoustic response in terms of electrical energy can be expressed as[6]

$$R_{pe} = \int_A p^2(x,y,z)\mathrm{d}A \cdot \frac{\eta_{pe}}{Z_a} \quad \text{(S-4)}$$

where $p(x,y,z)$ represents the complex amplitude of the pressure wave at a designated position (x,y,z), A denotes the acoustically sensitive area, η_{pe} is the acoustic-to-electrical conversion efficiency, and $Z_a = 1.5 \times 10^6$ Rayls is the acoustic impedance of the medium. In a focused

manner, Eq. (S-4) can be simplified as $R_{pe} = E_a \cdot \eta_{\mathrm{pe}}$, where $E_a = \frac{p^2 \cdot A}{Z_a}$ represents the incident acoustic energy. The transduction ratio is relatively low, with η_{pe}=0.001 to 0.01. The detection sensitivity can be scaled up by increasing the ultrasonically sensitive area A (or acoustic numerical aperture), but a bulky ultrasound sensor is not suitable for endoscopic applications.

B. Passive optical sensors

Figure S4b shows a typical sensing configuration based on an optical resonator. The acoustic pressure p modulates the round-trip optical path length L_{opt} and induces a frequency shift of the resonance spectrum. Here, an external laser source with a lasing frequency ω_{L} fixed at the resonance dip with the maximal slope is used to translate the acoustic modulation into a detectable optical intensity change, which is expressed by[7, 8]

$$S_p = \frac{\mathrm{d}I}{\mathrm{d}p} = I_0 \cdot \frac{\mathrm{d}T}{\mathrm{d}\omega_0} \cdot \frac{\mathrm{d}\omega_0}{\mathrm{d}L_{\mathrm{opt}}} \cdot \frac{\mathrm{d}L_{\mathrm{opt}}}{\mathrm{d}p} \tag{S-5}$$

where I_0 denotes the input optical intensity. The last two terms $\frac{\mathrm{d}L_{\mathrm{opt}}}{\mathrm{d}p}$ and $\frac{\mathrm{d}\omega_0}{\mathrm{d}L_{\mathrm{opt}}}$ have the same definitions as Eqs. (1) and (2) in the main text. The term $\frac{\mathrm{d}T}{\mathrm{d}\omega_0}$ measures the maximal spectral slope of the resonator. We write the transmission as a Lorentz function of angular frequency

$$T(\omega) = \frac{1}{1+\left(\frac{2\Delta\omega}{\Delta\omega_{\mathrm{FWHM}}}\right)^2} \tag{S-6}$$

where $\Delta\omega$ represents the frequency detuning and $\Delta\omega_{\mathrm{FWHM}}$ is the full spectral width at half maximum. Based on fundamental interferometry theory, a high-quality resonator has a maximal slope in the transmission spectrum with a detuning $\Delta\omega = \pm\frac{\sqrt{3}}{6}\Delta\omega_{\mathrm{FWHM}}$, and the corresponding slope is[7]

$$\left.\frac{\mathrm{d}T}{\mathrm{d}\omega_0}\right|_{\Delta\omega=-\frac{\sqrt{3}}{6}\Delta\omega_{\mathrm{FWHM}}} = \frac{3\sqrt{3}}{4} \cdot \frac{1}{\Delta\omega_{\mathrm{FWHM}}} \tag{S-7}$$

Using the definition of the quality factor $Q_c = \frac{\omega_{\mathrm{res}}}{\Delta\omega_{\mathrm{FWHM}}}$, Eq. (S-5) can be further expressed as

$$S_p = \frac{3\sqrt{3}}{4} I_0 \cdot Q_c \cdot S_m \tag{S-8}$$

When applying the monochromatic ultrasound wave with acoustic pressure $p(t) = \sin(\Omega t)$ to the sensor, the resonance frequency shifts by $\Delta\omega = p_0 S_{\mathrm{m}} \omega_0 \sin(\Omega t)$. Therefore, the output voltage at the photodetector can be expressed by

$$x(t) = x_o \left[1 + \frac{3\sqrt{3}}{4}\left(S_{\mathrm{m}} Q_c \sin(\Omega \mathrm{t})\right)\right] \tag{S-9}$$

where $x_o = I_0 H R$, where H (typical range: 0.6~0.8 A/W) denotes the optical-to-electrical conversion coefficient of the photodetector, and R is the resistor at the receiving end. As a result, the transduced electrical energy can be expressed as $E_t \cong \left(\frac{3\sqrt{3}}{4} I_0 \cdot Q_c \cdot S_m \cdot H\right)^2 \cdot R$, and the acoustic-to-electrical transduction ratio can be written as

$$\eta_{\mathrm{p}} = \frac{E_t}{E_a} = \frac{\frac{27}{16}(I_0 \cdot Q_{\mathrm{c}} \cdot S_{\mathrm{m}} \cdot H)^2 \cdot R}{\frac{A}{Z_{\mathrm{a}}}} \tag{S-10}$$

Eq. (S-10) suggests that the acoustic response can be significantly amplified by the quality factor Q_c of the resonator.

C. Optical heterodyne detection

The laser sensor shown in Fig. 1 and Fig. S1 has two polarized laser modes, expressed as $v_x = v_0 exp[i(\omega t + \varphi_x)] \cdot \vec{x}$ and $v_y = v_0 exp[i(\omega t + \varphi_y)] \cdot \vec{y}$, where $\varphi_{x,y}$ denotes the initial phase. An ultrasound wave induces an instantaneous phase change $\Delta\theta(t) = \pm S_{\mathrm{m}} G \cdot \sin(\Omega t)$ in the orthogonally polarized laser light beams. When the modulated light beams are injected at the photodetector, the obtained heterodyne signal can be expressed as

$$x(t) = x_o(1 + cos[\omega_b t + 2\Delta\theta(t) + \varphi_{\mathrm{b}}]) \quad \text{(S-11)}$$

where $\omega_{\mathrm{b}} = |\omega_x - \omega_y|$, $\varphi_{\mathrm{b}} = \varphi_x - \varphi_y$, $x_o = I_0 HR$, and $I_0 = v_0^2$. In the *I/Q* demodulation, the carrier frequency ω_b shifts to near *dc,* and the phase modulation is converted into a single-sideband signal[1]. Ignoring the *dc* component, in the narrow-band modulation condition, the output signal can be simplified as

$$x_{ac}(t) = 2x_o S_{\mathrm{m}} G \sin(\Omega t) \quad \text{(S-12)}$$

Therefore, the transduced electrical energy is $E_t = (I_0 \cdot G \cdot 2S_m \cdot H)^2 \cdot R$, and the acoustic-to-electrical transduction ratio for the laser sensor can be expressed as

$$\eta_{\mathrm{l}} = \frac{E_t}{E_a} = \frac{4(I_0 \cdot G \cdot S_{\mathrm{m}} \cdot H)^2 \cdot R}{\frac{A}{Z_{\mathrm{a}}}} \quad \text{(S-13)}$$

The similar expressions of η_{l} in Eq. (S-13) and η_{p} in Eq. (S-10) suggest that both passive optical and laser sensors can boost energy transduction. Table S1 lists the calculated energy transduction coefficients of some representative optical sensors based on Eqs. (S-10) and (S-13). For simplicity, we assume that the value of the term $(I_0 H)^2 \cdot R$ is 1 mW, which is typical for an undersaturated photodetector. We found that the coefficients of optical sensors can be significantly greater than 1, while piezoelectric sensors have a typical η_{pe} of 0.001 to 0.01. This explains why small-sized optical sensors can provide a detection sensitivity comparable to that of a focused piezoelectric sensor.

D. NEPDs of piezoelectric and optical sensors

The NEPD of the laser sensor was expressed as Eq. (4) in the main text. Here we derive the NEPD of a passive optical sensor. Based on the analysis in subsection *B*, the quadrature-phase noise component N_q corresponds to a lasing-frequency noise $N_q\Omega$ (based on the frequency-phase relation in the frequency domain)[1,2], resulting into an intensity noise $\frac{3\sqrt{3}}{4}\frac{N_q\Omega}{\Delta\omega_{\mathrm{FWHM}}}$. The noise power density can be expressed by $N = N_i + \frac{3\sqrt{3}}{4}\frac{N_q\Omega}{\Delta\omega_{\mathrm{FWHM}}}$, where N_i is the intensity noise power spectrum of the laser source. The corresponding NEPD can be expressed as

$$NEPD_{\mathrm{p}} = \frac{N}{S_{\mathrm{p}}} = \left(\frac{4\sqrt{3}N_i}{9Q_c} + \frac{N_q}{G}\right) \cdot \frac{1}{S_{\mathrm{m}}} \quad \text{(S-14)}$$

Assuming that $N_i = N_q = N_0$, which holds for many laser sources, it can be further written as

$$NEPD_{\mathrm{p}} = \frac{N_0}{S_{\mathrm{m}} G_{\mathrm{eq}}} \quad \text{(S-15)}$$

where G_{eq} is the equivalent gain factor defined by $\frac{1}{G_{\mathrm{eq}}} = \frac{4\sqrt{3}}{9Q_c} + \frac{1}{G}$. The quality factor Q_{c} of a resonator-based sensor ranges from 10^3 to 10^6, as listed in Tab. S1. In contrast, the gain factor G has a typical value of $\sim 2\times10^7$ at 10 MHz. As a result, we have $G_{\mathrm{eq}} \cong \frac{3\sqrt{3}}{4} Q_{\mathrm{c}}$ for most cases, and the laser sensor can have a much higher sensitivity gain than the passive sensor.

Figure S5 plots the sensor dimensions and NEPDs of the piezoelectric and optical sensors listed in Tab. S1, which suggests that optical sensors can have smaller sizes and high sensitivities. Therefore, optical sensors are favorable for PAE and other space-constrained imaging applications.

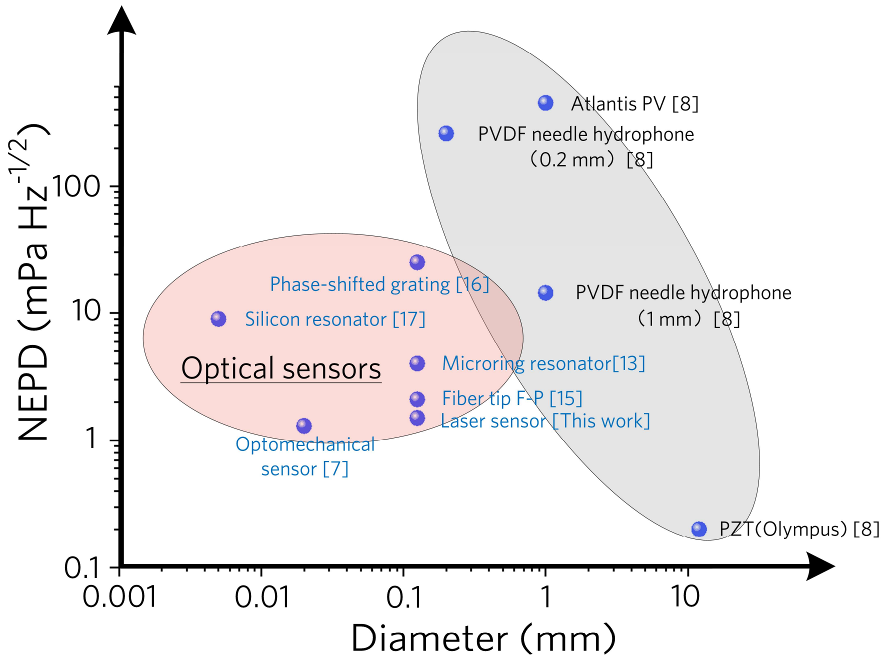

Supplementary Fig. S5. NEPDs of piezoelectric and optical ultrasound sensors versus diameter. Piezoelectric sensors degrade in sensitivity with decreasing sensor size. In contrast, optical sensors can offer sufficiently high sensitivities for hemodynamic imaging with sub-mm sizes and are suitable candidates for endoscopic imaging. Dashed line: Ideal relation between the NEPD and sensor diameter of piezoelectric sensors.

Supplementary Note S4: Error analysis of sO_2 measurement.

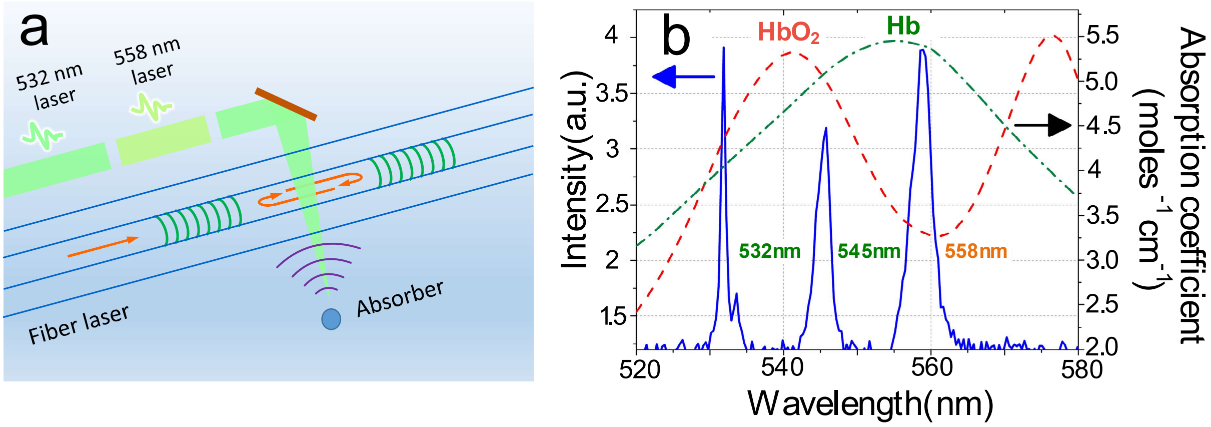

Supplementary Fig. S6. Photoacoustic measurement of sO_2. (a) Schematic of the experimental setup. The laser sensor detects photoacoustic signals induced by the absorption of dual-color laser pulses. sO_2 can be calculated based on the Hb/HbO_2 difference in the absorption coefficient at the two wavelengths. (b) Optical absorption spectra of oxy- and deoxygenated hemoglobin (dashed curves), and optical spectrum of the dual-wavelength laser source in the experiment. The 558-nm component is the second-order Stokes wave due to the SRS in a pure-silica-core optical fiber.

In biological and medical sciences, oxygen saturation (sO_2), the percentage of oxygenated hemoglobin with respect to total hemoglobin, is defined as[9, 10, 11]

$$\mathrm{sO_2} = \frac{C_{\mathrm{HbO_2}}}{C_{\mathrm{HbO_2}}+C_{\mathrm{Hb}}} \tag{S-16}$$

where $C_{\mathrm{HbO_2}}$ and C_{Hb} denote the concentrations of oxy- and deoxygenated hemoglobin (HbO_2 and Hb). Photoacoustic measurement of sO_2 takes advantage of the different absorption spectra of HbO_2 and Hb (Fig. S6a and b). Supposing that the laser spot size at a biological absorber is much smaller than the ultrasound wavelength, the excited photoacoustic wave strength is almost proportional to the absorption coefficient. Thus, by using multiple wavelength excitation, the measured hemoglobin spectrum can be known, and sO_2 can be calculated via spectral unmixing. Practically, a dual-wavelength laser source is used for subsequent photoacoustic excitation. The excited photoacoustic signals are expressed as

$$PA_1 = I_1 \cdot \mu_1^{HbO_2} \cdot C^{HbO_2} + I_1 \cdot \mu_1^{Hb} \cdot C^{Hb} \tag{S-17a}$$

$$PA_2 = I_2 \cdot \mu_2^{HbO_2} \cdot C^{HbO_2} + I_2 \cdot \mu_2^{Hb} \cdot C^{Hb} \tag{S-17b}$$

where μ denotes the absorption coefficient at each wavelength, C represents the molecular hemoglobin concentration, and I is the optical fluence. For simplicity, we assume that the optical fluences are identical at each wavelength.

Based on Eq. (S-17), we can calculate sO_2 from measured photoacoustic signals based on

$$\mathrm{sO_2} = \frac{\frac{PA_2}{PA_1} \cdot \mu_1^{Hb} - \mu_2^{Hb}}{\frac{PA_2}{PA_1} \cdot \Delta\mu_1 - \Delta\mu_2} \tag{S-18}$$

where $\Delta\mu_{1,2} = \mu_{1,2}^{Hb} - \mu_{1,2}^{HbO_2}$ is the Hb/HbO2 difference in the absorption coefficient at each wavelength. The measured photoacoustic strength has an error around the actual value in real-world measurements. This error can be transduced to sO_2 quantification, which can be estimated by calculating the first-order derivation of Eq. (S-18), expressed as

$$\delta sO_2 = \frac{|\Delta\mu_1 \cdot \mu_2^{Hb} - \Delta\mu_2 \cdot \mu_1^{Hb}|}{\left(\frac{PA_2}{PA_1} \cdot \Delta\mu_1 - \Delta\mu_2\right)^2} \cdot \left(\frac{1}{PA_1} + \frac{PA_2}{PA_1^2}\right) \cdot \delta PA \quad \text{(S-19)}$$

Here, we have assumed that the uncorrelated errors have identical values $\delta PA_1 = \delta PA_2 = \delta PA$. Eq. (S-19) can be further expressed as

$$\delta sO_2 \cong \frac{2|\Delta\mu_1 \cdot \mu_2^{Hb} - \Delta\mu_2 \cdot \mu_1^{Hb}|}{(\Delta\mu_1 - \Delta\mu_2)^2} \cdot \frac{1}{\text{SNR}} \quad \text{(S-20)}$$

where the signal-to-noise ratio $\text{SNR} = \frac{PA_{1,2}}{\delta PA}$, and we ignore the difference between PA_1 and PA_2 for simplicity. We used a 532- and 558-nm dual-wavelength laser for photoacoustic excitation in the experiment. Substituting μ_1^{Hb}=40584, μ_2^{Hb} = 54164, $\mu_1^{HbO_2}$ = 43876, and $\mu_2^{HbO_2}$ = 33456 ($cm^{-1} \cdot mole^{-1}$) into Eq. (S-20), we find that the noise is amplified by a factor of four in the transduction. As a result, quantitative assessment of sO_2 demands a high SNR of the photoacoustic measurement. For example, sO_2 measurements with an error of less than 10% (to distinguish arteries from veins) demand an SNR above 20 dB based on Eq. (S-20).

Supplementary Note S5: PAE.

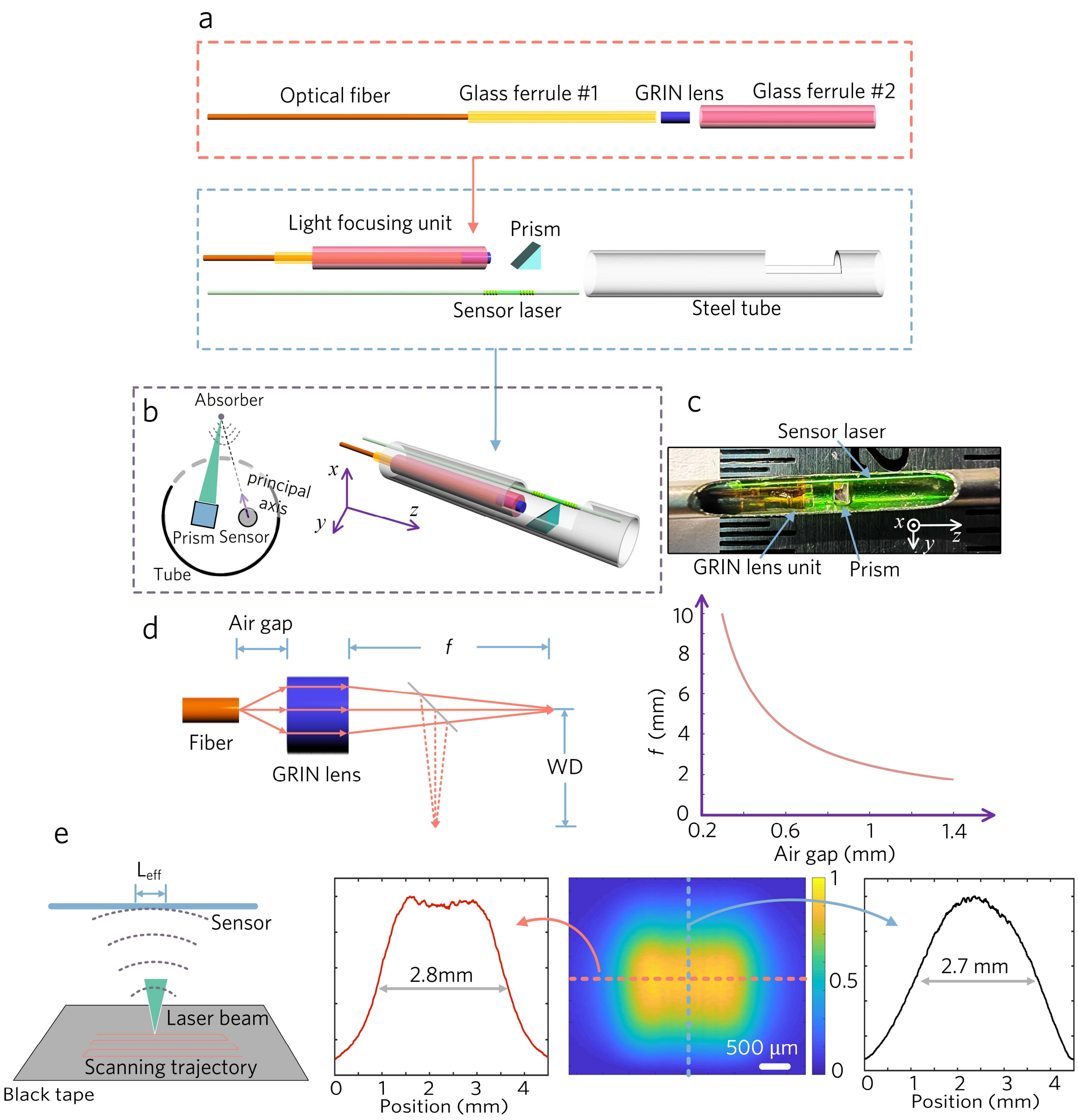

Supplementary Fig. S7. Fabrication of the endoscopic probe. (a) Assembly process, (b) schematic, and (c) photograph of the endoscopic probe. (d) Adjustment of focal length f by changing the air gap distance between the fiber endface and the GRIN lens. (e) Characterization of the spatial sensitivity of the laser sensor. The measured response is normalized to the maximal sensitivity.

A. Endoscopic probe

Figure S7a, b, and c shows the assembly process, a schematic, and a photograph of the

photoacoustic probe. A polarization-maintaining single-mode optical fiber (HB450-SC, Fibercore, N. A.: 0.14, mode field diameter: 3.5 μm) was used to deliver the excitation laser pulses to the probe. The fiber has a pure silica core to minimize the photodarkening effect. The optical fiber was first enclosed in glass ferrule #1 (inner diameter: 0.2 mm, outer diameter: 0.45 mm) to match the lens diameter. Then, the GRIN lens (GT-LFRL-050-024-20-NC, pitch: 0.24, length 2.94 mm, outer diameter: 0.5 mm, Grintech GmbH) were aligned with the fiber and encapsulated in ferrule #2 (inner diameter: 0.5 mm, outer diameter: 0.66 mm) to form the light focusing unit. As shown in Fig. S7d, the pulsed laser beam diverges from the fiber endface, passes through an air gap (approximately 0.5 mm), and then converges after passing through the GRIN lens. A Zemax simulation suggests that the focal length can be adjusted by changing the gap distance (Fig. S7d). We then placed a right-angle reflective prism (dimensions: 1 mm by 1 mm) in front of the GRIN lens endface to redirect the focused light beam to the biological tissue. The distance between the prism and GRIN lens was approximately 1.5 to 2 mm. For rectal imaging, we set a 2.7-mm optical working distance (WD), i.e., the distance from the tissue surface to the prism. After the WD was determined, all the components in the light focusing unit were fixed by ultraviolet curable adhesive (Optical adhesive 81, Norland). Next, we used another two glass ferrules (inner diameter: 0.3 mm, outer diameter: 0.5 mm, not shown in the figure) to enclose the pigtailed fibers of the sensor laser for structural support, leaving a bare laser cavity for ultrasound detection. The sensor laser, light focusing unit and prism were then fixed in a rigid stainless steel (SUS) tube as an endoscopic probe. The tube had a length of 105 mm and an outer diameter of 2 mm. A 10 mm-long, 180-degree wide window was created to transmit the optical and acoustic beams. Notably, the optical fiber section that emits green fluorescent light in Fig. S7c is the rare-earth-doped fiber. The fluorescent emission results from the energy transition from the $^4S_{3/2}$ to $^4I_{15/2}$ levels. The fluorescent light does not affect the 1550-nm laser output or the ultrasound detection.

We characterized the spatial sensitivity of the laser ultrasound sensor with the experimental setup shown in Fig. S7e. Here, we placed black tape 2.7 mm below the sensor and focused 532-nm laser pulses on it to generate ultrasound signals. The sensor received the laser-induced ultrasound waves while the laser focal spot was raster scanned. We rotated the principal axis of the sensor laser to be vertical to the tape in advance. Figure S7e shows the measured profile of the ultrasound sensitivity over the scanning plane. The flat-top profile in the longitudinal direction (along the laser sensor) follows the intracavity laser intensity distribution. In the orthogonal direction, the profile is determined by the incident angle of the ultrasound wave relative to the principal axis. Based on the characterization result, we aligned the light focusing unit and the laser sensor so that the focal spot of the pulsed laser fell in the central region of the color map to maximize the excitation-detection efficiency. We then fixed all the components in the probe by adhesive. Notably, only the normal incidence components of the spherical ultrasound wave can effectively induce an acoustic response of the sensor. The effects of the oblique components are canceled due to the phase variation of the acoustic wave along the fiber. As a result, the sensor receives only a small fraction of acoustic energy. The effective interaction length L_{eff} is determined by the acoustic wavelength and the distance to the acoustic source [5]. Despite the unfocused receiving manner, the sensor can provide sufficient sensitivity in PAE. The mismatch between the sensor laser length and L_{eff} can be minimized by using shorter laser cavities. Minimization of the cavity length depends on further increasing the optical gain of the rare-earth-doped fiber.

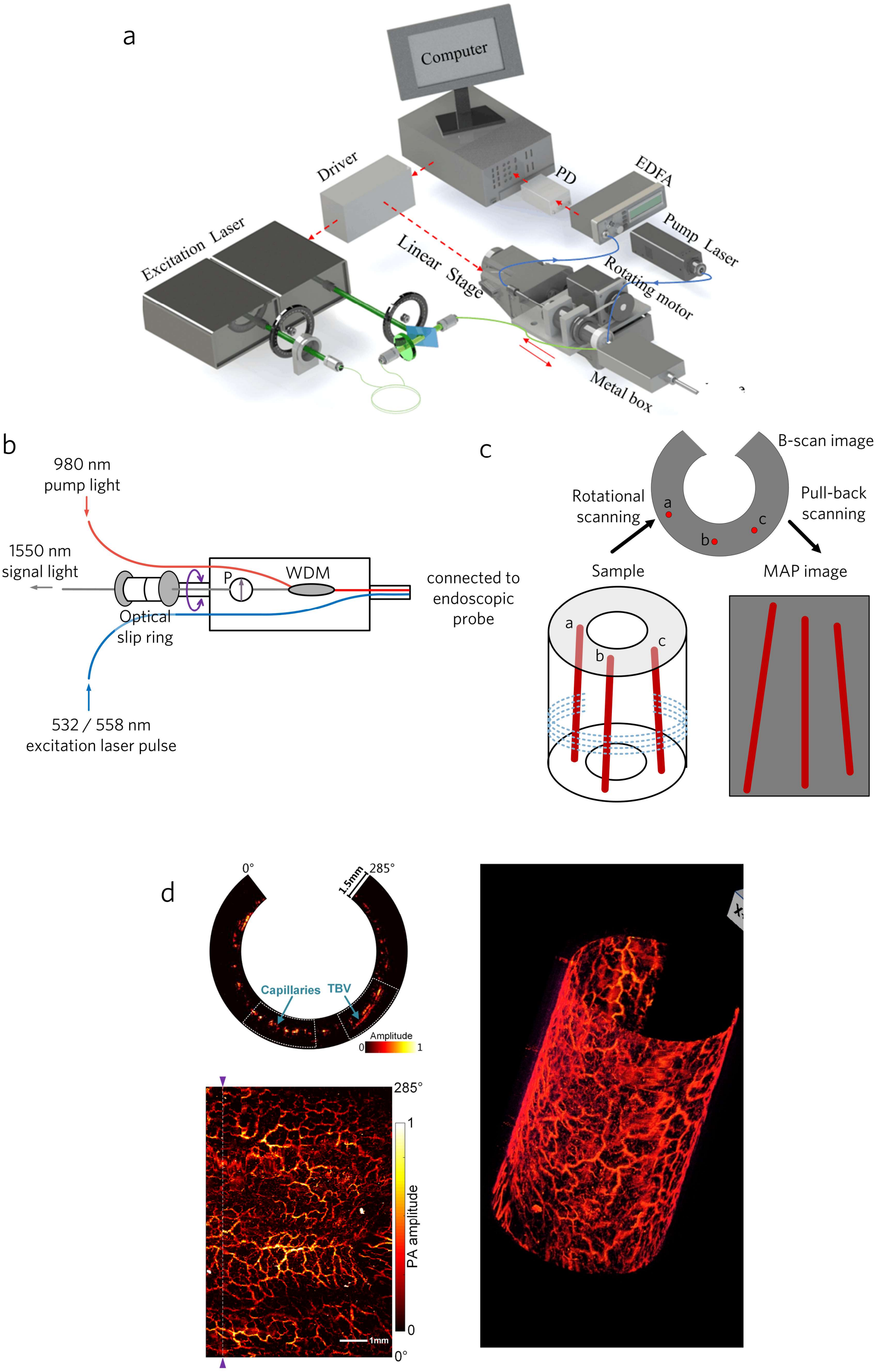

(to be continued)

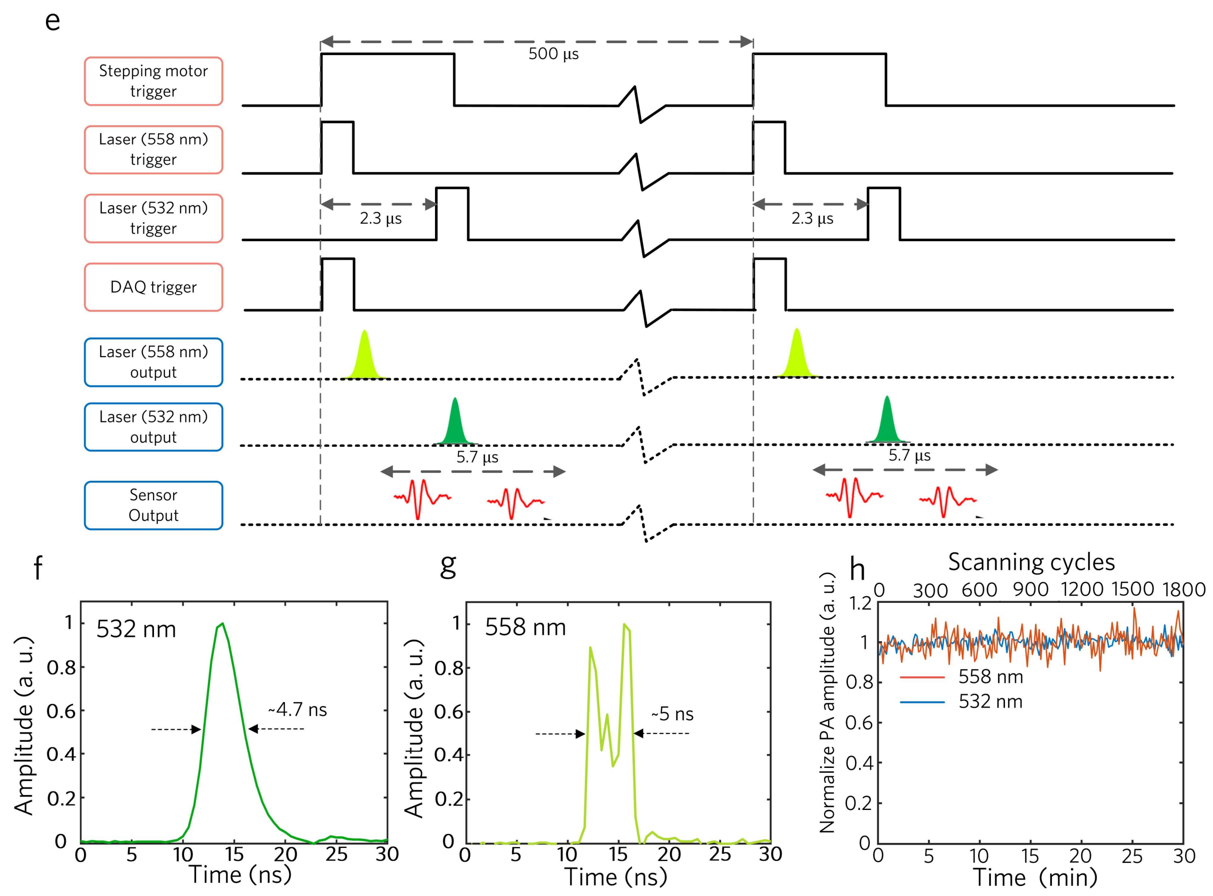

Supplementary Fig. S8. PAE system. (a) Experimental setup. (b) Schematic of the rotational scanning unit. (c) Schematic of the endoscopic scanning imaging. (d) PAE imaging result, including a B-scan image (upper left), a MAP image (lower left), and a 3D-rendered image (right) from a rat rectum. Measured waveforms of the 532-nm (f) and 558-nm (g) laser pulses. (h) Stability test result of photoacoustic signals excited by the two wavelengths. PD: photodetector. EDFA: erbium-doped fiber amplifier. WDM: 980/1550 nm wavelength-division multiplexer. TBV: trunk blood vessel.

B. PAE

Figure S8a shows the experimental setup for PAE. The system consists of a photoacoustic probe, a dual-wavelength laser source, a sensor interrogation unit, mechanical scanners, and DAQ and control modules. As shown in Fig. S8b, a 980/1550 nm WDM and a polarizer were encapsulated in a metal box, which rotated with the endoscopic probe. To minimize polarization fluctuations, we used a polarization-maintaining fiber to guide the signal light (1550 nm). An optical slip ring connected the signal output to the photodetector. As shown in Fig. S8c, the photoacoustic probe delivers a pair of dual-wavelength laser pulses into biological tissue, detects the optically induced ultrasound waves, and forms an A-line. Each A-line contains 800 points. We interleaved the 532- and 558-nm pulses by 2.3 μs to avoid overlap of the subsequent photoacoustic signals or induction of the Grueneisen relaxation effect. The probe was rotated back and forth using a rotation stage to form a B-scan. We used 2000 A-lines to create a B-scan image, with a

0.11° step size. The B-scan rate was 1 Hz. Then, a linear stage pulled the probe back step by step to produce a three-dimensional image. The pullback step was 16 µm, and the speed was ~15 µm/s. Finally, 2000 B-scan slices were acquired to form a C-scan. Figure S8d shows a typical PAE image from a rat rectum (Sprague Dawley rat, female, 250 g) obtained at 532 nm. The B-scan image exhibits an angular field of view of 285°, which is the maximal value that we can achieve thus far (210° in the main text). We maintained anesthesia with a 1.5–2.0% isoflurane supply during the imaging. The maximum amplitude projection (MAP) and 3D-rendered images exhibit the trunk blood vessels (TBVs) and capillaries at the rectum wall. We further achieved functional imaging using dual-wavelength excitation, as shown in Figs. 3 and 4 in the main text. Notably, Fig. 3 in the main text shows some unexpected bright spots in region #1, as a result from the imperfect rectal wall cleaning. To avoid direct contact between the rotational scanning probe and biological tissue, we used a stainless steel tube (inner diameter: 4 mm, outer diameter: 5.5 mm) as a sheath. A side window was produced to allow transmission of the laser beam and photoacoustic signals during the rotational scanning. We covered this window with an acoustically and optically transparent polyethylene terephthalate (PET) membrane. We then filled the sheath tube with deionized water as an acoustic coupling medium.

Figure S8e shows the timing charts of the imaging system. We used a control card (PXI-7852R, National Instruments) to synchronize the laser pulses, motorized stages, and DAQ. It operated at 2 kHz, following the laser pulse repetition rate. Figure S8f and g shows the waveforms of the 532-nm and 558-nm laser pulses, measured by using a high-speed photodetector (DET025A, Thorlabs) with a 1.8-GHz sampling rate. The pulse widths are 4.7 and 5.0 ns, respectively. The 558-nm pulse waveform changes to a flat-top or a dual-peak profile in the nonlinear wavelength conversion process [11]. However, this variation induces an amplitude change in the high-frequency components of the photoacoustic signal, which is hardly detectable by the laser sensor. As a result, the sO_2 measurement is hardly affected[12]. Figure S8h shows the stability test result of the photoacoustic signals excited by the two wavelengths over 30 min. Although the 558-nm laser output presents a more noticeable amplitude fluctuation, it is still acceptable for sO_2 quantification.

Table S1. Ultrasound sensitivities and energy transduction ratios of piezoelectric and optical sensors.

Sensor type	Optical sensors							Piezoelectric sensors	
	Microring resonator[13]	Fabry–Perot cavity (planar)[14]	Fabry–Perot cavity (fiber)[15]	Optomechanical waveguide[7]	Phase-shifted Bragg grating[16]	Silicon nanoresonator[17]	Laser sensor *(this work)*	Spherically focused piezoceramic[8]	PVdF needle hydrophone[8]
Quality factor* ($\times 10^6$)	0.14	0.0028	0.1	0.003	1.2	0.02	10 (at 20 MHz)	N. A.	N. A.
S_m ($\times 10^{-6}$ MPa^{-1})	130	90	100	19354	3.8	5	5.5	N. A.	N. A.
Sensing area *A* ($\times 10^{-3}$ mm^2)	0.32	6.4	0.2	0.32	2.7	0.0001	10	30000	1000
Transduction efficiency η (mm^{-2})	2621	0.025	1266	26668	20	253	1815	0.01	0.001
NEPD (mPa·Hz$^{-1/2}$)	5.6	78	2.1	1.3	25	9	1.5	0.2	14.4

* Here, we list the value of the physical quality factor of the optical resonators Q_c for the former six works and *G* for the laser sensor, which are associated with the sensitivity gain in energy transduction.

References for Supplementary Materials

1. Ziemer RE, Tranter WH. *Principles of communications*. John Wiley & Sons (2014).

2. Rønnekleiv E. Frequency and intensity noise of single frequency fiber Bragg grating lasers. *Optical Fiber Technology* **7**, 206-235 (2001).

3. Cranch G, Miller G. Fundamental frequency noise properties of extended cavity erbium fiber lasers. *Optics letters* **36**, 906-908 (2011).

4. Urick VJ, Williams KJ, McKinney JD. *Fundamentals of microwave photonics*. John Wiley & Sons (2015).

5. Bai X*, et al.* Sensitivity characteristics of broadband fiber-laser-based ultrasound sensors for photoacoustic microscopy. *Optics Express* **25**, 17616-17626 (2017).

6. Winkler AM, Maslov KI, Wang LV. Noise-equivalent sensitivity of photoacoustics. *Journal of biomedical optics* **18**, 097003 (2013).

7. Westerveld WJ*, et al.* Sensitive, small, broadband and scalable optomechanical ultrasound sensor in silicon photonics. *Nature Photonics* **15**, 341-345 (2021).

8. Wissmeyer G, Pleitez MA, Rosenthal A, Ntziachristos V. Looking at sound: optoacoustics with all-optical ultrasound detection. *Light: Science & Applications* **7**, 1-16 (2018).

9. Perekatova V, Subochev P, Kleshnin M, Turchin I. Optimal wavelengths for optoacoustic measurements of blood oxygen saturation in biological tissues. *Biomedical Optics Express* **7**, 3979-3995 (2016).

10. Allen TJ, Ogunlade O, Zhang E, Beard PC. Large area laser scanning optical resolution photoacoustic microscopy using a fibre optic sensor. *Biomedical optics express* **9**, 650-660 (2018).

11. Liang Y, Jin L, Guan B-O, Wang L. 2 MHz multi-wavelength pulsed laser for functional photoacoustic microscopy. *Optics letters* **42**, 1452-1455 (2017).

12. Liang Y, Liu H, Li Q, Jin L, Guan B-O, Wang L. Acoustic-spectrum-compensated photoacoustic microscopy. *Optics Letters* **45**, 1850-1853 (2020).

13. Zhang C, Chen S-L, Ling T, Guo LJ. Review of imprinted polymer microrings as ultrasound detectors: Design, fabrication, and characterization. *IEEE Sensors Journal* **15**, 3241-3248 (2015).

14. Zhang E, Laufer J, Beard P. Backward-mode multiwavelength photoacoustic scanner using a planar Fabry-Perot polymer film ultrasound sensor for high-resolution three-dimensional imaging of biological tissues. *Applied optics* **47**, 561-577 (2008).

15. Guggenheim JA*, et al.* Ultrasensitive plano-concave optical microresonators for ultrasound sensing. *Nature Photonics* **11**, 714-719 (2017).

16. Ülgen O, Shnaiderman R, Zakian C, Ntziachristos V. Interferometric optical fiber sensor for optoacoustic endomicroscopy. *Journal of Biophotonics*, e202000501 (2021).

17. Shnaiderman R, Wissmeyer G, Ülgen O, Mustafa Q, Chmyrov A, Ntziachristos V. A submicrometre silicon-on-insulator resonator for ultrasound detection. *Nature* **585**, 372-378 (2020).
