# Supplementary material for: Optical-resolution functional gastrointestinal photoacoustic endoscopy based on optical heterodyne detection of ultrasound
Source: Nat Commun. 2022 Dec 9;13:7604. doi: 10.1038/s41467-022-35259-5 (PMC9734171; doi:10.1038/s41467-022-35259-5)
Supplement: Supplementary file 2 — Reporting Summary [file 41467_2022_35259_MOESM2_ESM.pdf]

nature portfolio

Corresponding author(s): Long Jin

Last updated by author(s): Oct 25, 2022

Reporting Summary

Nature Portfolio wishes to improve the reproducibility of the work that we publish. This form provides structure for consistency and transparency in reporting. For further information on Nature Portfolio policies, see our Editorial Policies and the Editorial Policy Checklist.

Statistics

For all statistical analyses, confirm that the following items are present in the figure legend, table legend, main text, or Methods section.

n/a	Confirmed	
☐	☒	The exact sample size (*n*) for each experimental group/condition, given as a discrete number and unit of measurement
☐	☒	A statement on whether measurements were taken from distinct samples or whether the same sample was measured repeatedly
☐	☒	The statistical test(s) used AND whether they are one- or two-sided<br>*Only common tests should be described solely by name; describe more complex techniques in the Methods section.*
☒	☐	A description of all covariates tested
☒	☐	A description of any assumptions or corrections, such as tests of normality and adjustment for multiple comparisons
☒	☐	A full description of the statistical parameters including central tendency (e.g. means) or other basic estimates (e.g. regression coefficient) AND variation (e.g. standard deviation) or associated estimates of uncertainty (e.g. confidence intervals)
☐	☒	For null hypothesis testing, the test statistic (e.g. *F*, *t*, *r*) with confidence intervals, effect sizes, degrees of freedom and *P* value noted<br>*Give P values as exact values whenever suitable.*
☒	☐	For Bayesian analysis, information on the choice of priors and Markov chain Monte Carlo settings
☒	☐	For hierarchical and complex designs, identification of the appropriate level for tests and full reporting of outcomes
☒	☐	Estimates of effect sizes (e.g. Cohen's *d*, Pearson's *r*), indicating how they were calculated

Our web collection on statistics for biologists contains articles on many of the points above.

Software and code

Policy information about availability of computer code

Data collection	The authors declare that for data collection the commercially available software from LabVIEW 2015 (National Instruments, USA)。
Data analysis	Data analysis was conducted in Matlab 2018b (Matlab, Mathworks, USA) using its built-in functions. The image algorithm has been patented and is available upon discretion from the corresponding author.

For manuscripts utilizing custom algorithms or software that are central to the research but not yet described in published literature, software must be made available to editors and reviewers. We strongly encourage code deposition in a community repository (e.g. GitHub). See the Nature Portfolio guidelines for submitting code & software for further information.

Data

Policy information about availability of data

All manuscripts must include a data availability statement. This statement should provide the following information, where applicable:

- Accession codes, unique identifiers, or web links for publicly available datasets
- A description of any restrictions on data availability
- For clinical datasets or third party data, please ensure that the statement adheres to our policy

The raw signal and noise data for calibration of the laser sensor, the raw image data of of mice and rats that were generated in this study have been deposited in the Zenodo database under accession code:

Human research participants

Policy information about studies involving human research participants and Sex and Gender in Research.

Reporting on sex and gender	We did not perform any human participants research.
Population characteristics	We did not perform any human participants research.
Recruitment	We did not perform any human participants research.
Ethics oversight	We did not perform any human participants research.

Note that full information on the approval of the study protocol must also be provided in the manuscript.

Field-specific reporting

Please select the one below that is the best fit for your research. If you are not sure, read the appropriate sections before making your selection.

[x] Life sciences [ ] Behavioural & social sciences [ ] Ecological, evolutionary & environmental sciences

For a reference copy of the document with all sections, see nature.com/documents/nr-reporting-summary-flat.pdf

Life sciences study design

All studies must disclose on these points even when the disclosure is negative.

Sample size	No statistical method was used to predetermine sample size（Two mice and four rats were imaged in this study）,because the biological samples were used only as a vehicle to demonstrate a novel imaging approach
Data exclusions	No data were excluded intentionally.
Replication	Replicate experiments were successful. The exact number of replicates of each experiments is given in the figure legends and in the manuscripts.
Randomization	Randomization was not relevant to our study because no treatments were involved.
Blinding	Blinding was not relevant to our study because there was only one experimental group.

Reporting for specific materials, systems and methods

We require information from authors about some types of materials, experimental systems and methods used in many studies. Here, indicate whether each material, system or method listed is relevant to your study. If you are not sure if a list item applies to your research, read the appropriate section before selecting a response.

Materials & experimental systems

n/a	Involved in the study	
[x]	[ ]	Antibodies
[x]	[ ]	Eukaryotic cell lines
[x]	[ ]	Palaeontology and archaeology
[ ]	[x]	Animals and other organisms
[x]	[ ]	Clinical data
[x]	[ ]	Dual use research of concern

Methods

n/a	Involved in the study	
[x]	[ ]	ChIP-seq
[x]	[ ]	Flow cytometry
[x]	[ ]	MRI-based neuroimaging

Animals and other research organisms

Policy information about studies involving animals; ARRIVE guidelines recommended for reporting animal research, and Sex and Gender in Research

Laboratory animals	Both the mice and rat were housed with 12-h light/dark cycle at 25 °C and free access to food and water. 2 months old male BALB/c mouse and 2 months old male Sprague Dawley rat were use.

Wild animals	We did not use any wild animals.
Reporting on sex	Sex was not considered in the experiment.
Field-collected samples	We did not use any field-collected samples.
Ethics oversight	All procedures were conducted in“Guiding Principles in the Care and Use of Animals” (GB/T 35892-2018,China) and were approved by the Laboratory Animal Ethics Committee of Guangzhou Huateng Biomedical Technology(IACUC:HTSW220304)

Note that full information on the approval of the study protocol must also be provided in the manuscript.
